# The Impact of Nutritional Status on Survival and Development of Sarcoidosis: A Scoping Review of Current Evidence and Research Gaps

**DOI:** 10.3390/nu18020209

**Published:** 2026-01-09

**Authors:** Jacek Kobak, Angelika Szymańczyk, Martyna Liśkiewicz-Jankowska, Monika Cichoń-Kotek, Mateusz Szczupak

**Affiliations:** 1 Department of Otolaryngology, Faculty of Medicine, Medical University of Gdańsk, 80-210 Gdańsk, Poland; 2 Department of Otolaryngology, University Clinical Center, 80-214 Gdańsk, Poland; 3 Department of Anesthesiology and Intensive Care, Copernicus Hospital in Gdańsk, 80-803 Gdańsk, Poland; angelika.szymanczyk@gmail.com (A.S.); szczupak.mateusz@icloud.com (M.S.); 4 Department of Hypertension and Diabetology, Faculty of Medicine, Medical University of Gdańsk, 80-214 Gdańsk, Poland; martyna.jankowska@gumed.edu.pl; 5 Department of Pediatric, Hematology and Oncology, Faculty of Medicine, Medical University of Gdańsk, 80-214 Gdańsk, Poland; m.cichon@gumed.edu.pl

**Keywords:** sarcoidosis, nutritional status, obesity, calcium metabolism, inflammation, prognosis

## Abstract

**Background**: Sarcoidosis is a heterogeneous, multisystem inflammatory disease with an unpredictable clinical course and limited prognostic markers. Increasing attention has focused on nutritional and metabolic factors—particularly obesity, body composition, and calcium–vitamin D metabolism—as potentially modifiable elements associated with disease development and clinical phenotype. However, the available literature remains fragmented and methodologically heterogeneous. **Objective**: To systematically map current evidence on the relationship between nutritional status and the development, clinical course, and prognosis of sarcoidosis, and to identify key gaps requiring further research. **Methods**: A scoping review was conducted in accordance with the Joanna Briggs Institute methodology and the PRISMA-ScR guidelines. PubMed, Scopus, Web of Science, Cochrane Library, EBSCO, and Google Scholar were searched for studies published between 2015 and 2025. Eligible studies included adult patients with sarcoidosis and addressed nutritional status broadly defined, encompassing anthropometric measures, body composition, immunonutritional indices, nutrition-related biomarkers, dietary factors, and supplementation practices. Due to substantial heterogeneity in exposure definitions and outcome measures, no quantitative synthesis or formal methodological quality appraisal was performed. **Results**: Eighteen studies, predominantly observational, were included. The most consistent findings concerned anthropometric parameters, with overweight and obesity showing the strongest association with an increased risk of sarcoidosis and, in selected studies, with reduced exercise capacity and greater disease burden. Evidence linking nutritional status to prognosis was indirect, while direct data on sarcoidosis-specific survival were lacking. Disturbances in calcium–vitamin D metabolism were frequent and clinically relevant, particularly in the context of supplementation-related hypercalcemia. **Conclusions**: Current evidence suggests that nutritional status—particularly excess body weight—and selected metabolic and immunonutritional factors are associated with sarcoidosis. However, given the largely observational nature of the available data and the lack of formal assessment of methodological quality, these results should be interpreted as association mapping and hypothesis generation rather than as evidence of causality. Well-designed prospective and interventional studies using standardized nutritional assessment tools and clinically relevant endpoints are needed to clarify the role of nutritional factors in sarcoidosis.

## 1. Introduction

Sarcoidosis is a chronic inflammatory disease of unknown etiology, characterized by the formation of non-caseating granulomas in the tissues of affected organs, most commonly in the lungs and mediastinal lymph nodes, but also in extrapulmonary organs such as the skin, eyes, liver, heart, and central nervous system [1,2,3]. The etiopathogenesis of sarcoidosis is complex and involves genetic, immunological, and potential environmental factors, but the specific antigen that initiates the inflammatory process has not been clearly identified [3]. Inflammation in sarcoidosis results from the activation of both the innate and adaptive immune responses, leading to the accumulation of T lymphocytes and the differentiation of macrophages into granulomatous types, maintaining chronic inflammation [4].

Sarcoidosis exhibits considerable clinical and prognostic heterogeneity, which means that its clinical presentation, course, and prognosis can vary significantly between patients. Although the lungs and lymph nodes are the most commonly affected organs, the clinical symptoms of the disease depend on the location and severity of the lesions. They may include both general symptoms, such as fatigue, fever, night sweats, and weight loss, as well as organ-specific symptoms, including cough, shortness of breath, joint pain, skin lesions, visual disturbances, or cardiac or neurological symptoms [3,5,6]. Symptomatic heterogeneity means the disease may be asymptomatic and detected incidentally on imaging studies, or it may cause severe organ dysfunction with a significant impact on the patient’s functioning [7].

The course of sarcoidosis can also be unpredictable. In a significant proportion of patients (approximately 50–70%), the disease is self-limiting, with remission of symptoms within 12–36 months of diagnosis, while in 10–30% of patients, the course becomes chronic and requires long-term treatment, often immunosuppressive. The course’s diversity and the lack of clear prognostic markers make assessing the risk of progression and response to therapy a clinical challenge. Demographic and clinical factors influence the course of the disease; for example, in some ethnic populations, involvement of organs such as the heart and brain is associated with a poorer prognosis and a higher risk of complications [7].

The clinical significance of sarcoidosis stems both from its multi-organ nature and its potential impact on patients’ quality of life and functioning. In some patients, persistent fatigue, shortness of breath, pain, and limited physical activity can lead to permanent disability, even if the disease does not progress to severe organ complications [3].

A particularly clinically significant subtype is sarcoidosis-associated pulmonary fibrosis (SAPF), which develops in a smaller number of patients but is associated with higher morbidity [8]. Pulmonary fibrosis is a key turning point in the disease. In advanced forms (often corresponding to radiological stage IV), the prognosis may be determined by the extent of fibrotic changes and vascular complications, including sarcoidosis-associated pulmonary hypertension (SAPH), which is one of the most important factors increasing the risk of death [9,10]. For this reason, in clinical practice, it is essential not only to diagnose sarcoidosis but also to monitor organ involvement, assess disease activity and prognosis, and tailor therapy to the individual patient’s risk of progression.

Despite growing interest in the nutrition-immunometabolism axis and reports suggesting a link between metabolic parameters (including obesity) and sarcoidosis risk, the literature remains scattered and methodologically heterogeneous, and the concept of nutritional status is often operationalized in ways that are not comparable across studies. There is a lack of mapping of the nutritional and metabolic exposures analyzed to date, the tools used to measure them, and the disease endpoints to which they have been linked. As a result, it is unclear which areas require standardization of measurement, which populations are underrepresented, and where there is potential for prospective and interventional studies. A scoping review is an appropriate approach to organizing this field, identifying key concepts, and precisely identifying research gaps that form the basis for future studies with higher evidence strength [11,12,13].

The aim of this scoping review was to systematically map the available scientific evidence on the relationship between nutritional status and the development, clinical course, prognostic indicators, and outcomes of sarcoidosis in patients. In particular, the aim was to identify the nutritional and metabolic exposures analyzed, the tools used to assess them, and the endpoints used in the studies. To date, this is the first scoping review to comprehensively map the evidence linking nutritional status to progression and prognosis in sarcoidosis, while also identifying key research gaps.

## 2. Interactions Between Nutrition, Inflammation, and Immune-Mediated Diseases

Nutritional status is one of the keys, potentially modifiable environmental factors affecting the functioning of the immune system and the course of chronic inflammatory diseases. The modern approach to nutrition goes beyond the classic perception of diet as a source of energy and building blocks, considering its role in regulating the immune response through immunometabolic mechanisms, modulating the gut microbiota, and influencing low-grade chronic inflammation [10,14].

One of the best-described mechanisms linking diet and immunity is the diet–gut microbiota–immune system axis. The composition and quality of the diet determine the diversity of the gut microbiota and the profile of its metabolites, which in turn modulate immune cell maturation, intestinal barrier integrity, and systemic inflammatory tone. Some of the best-characterized bacterial metabolites are short-chain fatty acids (SCFAs), such as butyrate, propionate, and acetate, which are produced by the fermentation of dietary fiber. SCFAs exhibit immunomodulatory properties by activating G protein-coupled receptors, inhibiting histone deacetylases, and influencing the differentiation of regulatory T cells, thereby promoting immune tolerance and reducing chronic inflammation [14,15,16,17,18,19].

Disorders of the microbiota composition (dysbiosis), observed, among others, in obesity and high consumption of highly processed foods, can lead to reduced SCFA production, increased intestinal barrier permeability, and translocation of pro-inflammatory signals into the systemic circulation. These mechanisms are increasingly recognized as part of the pathogenesis of inflammatory and autoimmune diseases. Although direct data on the gut microbiota in sarcoidosis are limited, this concept provides a biologically plausible background for the observed associations between diet, obesity, and disease activity [18,20].

In terms of dietary patterns, the Mediterranean diet is of particular interest, as numerous systematic reviews and meta-analyses have linked it to reduced levels of inflammatory markers such as CRP (C-reactive protein) and IL-6 (Interleukin-6), as well as a favorable metabolic profile. Its potential anti-inflammatory effect is attributed to its high fiber, polyphenol, and unsaturated fatty acid content, as well as its low intake of highly processed foods [21,22]. In contrast, a diet based on ultra-processed foods is associated with higher levels of inflammatory markers and may contribute to the perpetuation of low-grade chronic inflammation [23,24].

Obesity, currently considered a state of chronic low-grade inflammation, is also an important link between nutrition and immune response. Adipose tissue acts as an active endocrine and immune organ, producing numerous pro-inflammatory adipokines and cytokines that can modulate the immune response and promote inflammatory dysregulation [25]. In this context, the term immunometabolism is increasingly used to describe the interrelationships between metabolism and immune cell function in chronic diseases.

Selected nutrients with documented immunomodulatory properties also play an essential role. It has been shown that omega-3 fatty acid supplementation in adults can reduce markers of inflammation, such as CRP, IL-6, and TNF-α (tumor necrosis factor alpha). However, the effectiveness of this intervention depends on the clinical context, the dose used, and the duration of supplementation [26]. Regarding vitamin D, data from large retrospective meta-analyses indicate that supplementation may reduce the risk of acute respiratory infections under certain conditions (especially in deficient individuals), demonstrating the impact of nutritional status on immune function [27].

Concerning sarcoidosis, the above mechanisms provide a coherent, biologically plausible context for the observed associations between excess body weight, dietary factors, and the risk of developing the disease. Despite the lack of direct mechanistic studies, a growing body of indirect evidence supports the need for further research on the role of nutrition, gut microbiota, and immunometabolism in the pathogenesis and course of sarcoidosis.

**Figure 1:**
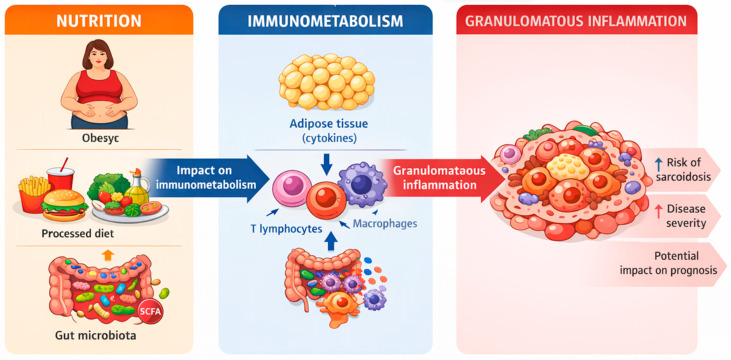
Nutrition-immunometabolism-granulomatous inflammation axis.

The above clinical observations can be interpreted in the context of the broader nutrition–immunometabolism–granulomatous inflammation axis (Figure 1), in which excessive adiposity, unfavorable body composition, and metabolic disorders may modulate immune system activation and the persistence of granulomatous inflammation.

## 3. Microbiota of the Gastrointestinal Tract and Respiratory Tract, SCFA, and Their Potential Significance in Sarcoidosis

Although the diet–microbiota–immune axis is well documented as a mechanism for modulating chronic inflammation, in sarcoidosis, direct evidence for the role of gut microbiota and metabolites, such as SCFA, remains limited. From a biological perspective, particular importance is attached to the so-called gut–lung axis (airway axis), in which gut microbiota metabolites (including SCFA) and microbial ligands can influence the maturation and polarization of immune responses in distant organs (airway axis), in which gut microbiota metabolites (including SCFA) and microbial ligands can influence the maturation and polarization of immune responses in distant organs, including the respiratory system, by modulating the function of dendritic cells, macrophages, and the Th17/Treg balance [28,29].

In the context of sarcoidosis, the most consistent empirical data concern the respiratory microbiome. An extensive cross-sectional study evaluating the lung microbiota found distinct microbiome composition in patients with sarcoidosis compared to healthy individuals, with an overrepresentation of selected taxa (including *Atopobium* and *Fusobacterium*), suggesting a potential link between respiratory tract dysbiosis and the granulomatous process [30,31]. In addition, specific microbiological “signatures” in the circulation (the so-called blood microbiome) have also been described in patients with sarcoidosis, including an increased abundance of, among others, *Veillonella*, *Prevotella*, *Cutibacterium*, *Corynebacterium*, and *Streptococcus*, which reinforces the hypothesis of the involvement of microbial stimuli in maintaining immune activation in some patients [32].

Evidence regarding the gut microbiota in sarcoidosis is less abundant. Still, studies suggest that specific microbiota profiles may correlate with the immune “landscape” and inflammatory phenotype (e.g., signatures associated with heme biosynthesis and monocyte activation), a potential mechanism linking diet-related factors, microbiota, and the granulomatous process [33].

Considering the above, mechanistic hypotheses about the role of SCFAs in sarcoidosis are currently biologically plausible but require further validation in prospective and interventional studies.

## 4. Analysis of Nutritional Status in Sarcoidosis

Nutritional status assessment in patients with sarcoidosis should be considered an integral part of clinical evaluation, both at the time of diagnosis and during ongoing monitoring. This is due to the multi-organ nature of sarcoidosis, its heterogeneous course, and the complex interactions among chronic inflammation, metabolism, and immune system function [34,35]. A growing body of evidence suggests that nutritional parameters may influence the risk of developing the disease, its clinical phenotype, physical performance, quality of life, and the safety of treatment [36].

Population and clinical studies have shown that overweight and obesity are significantly associated with an increased risk of developing sarcoidosis [35,37]. Adipose tissue functions as an active endocrine and immune organ, participating in the regulation of the inflammatory response through the production of pro-inflammatory adipokines and cytokines, which may promote chronic inflammation and modulate the granulomatous response [25]. For this reason, the assessment of nutritional status in patients with sarcoidosis should not be limited to body mass index (BMI) alone. Still, it should also include analysis of body composition and skeletal muscle function, especially in the context of limited physical activity and chronic fatigue, which often accompany the disease [36,38,39].

The contemporary approach to diagnosing malnutrition and nutritional disorders recommends the use of standardized criteria, such as the consensus of the Global Leadership Initiative on Malnutrition (GLIM). According to these recommendations, the diagnosis of malnutrition requires at least one phenotypic criterion (e.g., unintentional weight loss, low body weight, or reduced muscle mass) and one etiological criterion, which includes chronic inflammatory disease. Sarcoidosis, as a disease characterized by chronic systemic inflammation, meets the GLIM etiological criterion, which justifies systematic screening and in-depth nutritional assessment in this population [40].

From a clinical perspective, assessing body composition is particularly important, as patients with sarcoidosis may have excessive body fat while also experiencing reduced skeletal muscle mass and function. Studies have shown that poorer nutritional status, higher body fat levels, and unfavorable body composition are associated with reduced exercise tolerance, decreased muscle strength, and poorer physical performance in patients with sarcoidosis. In this context, it is reasonable to consider methods of body composition analysis, such as bioelectrical impedance analysis (BIA) or DXA densitometry, as well as simple functional tests (e.g., handgrip strength), to identify early signs of sarcopenia or sarcopenic obesity [38,41].

Another key element in analyzing nutritional status in sarcoidosis is the impact of pharmacological treatment, especially glucocorticosteroids (GCS) therapy, which remains the basis of treatment for many forms of the disease, and is associated with weight gain, insulin resistance, adverse changes in body composition, and the development of glucocorticosteroid-induced osteoporosis. Current clinical guidelines recommend systematic assessment of metabolic and bone risk in patients receiving chronic GCS, including nutritional and lifestyle factors as an integral part of a preventive strategy [42,43].

Sarcoidosis is also characterized by specific disturbances in calcium and vitamin D metabolism, which are directly related to nutritional and supplementation issues. Extrarenal activation of 1α-hydroxylase in granulomas leads to increased synthesis of the active form of vitamin D, which, in some patients, results in hypercalcemia and hypercalciuria [44]. This phenomenon poses a significant clinical challenge, as routine vitamin D supplementation, commonly used in the general population, may increase the risk of metabolic and renal complications in patients with sarcoidosis [45]. Current reviews and clinical positions emphasize the need to individualize decisions regarding vitamin D supplementation and to regularly monitor serum calcium levels and urinary calcium excretion in patients with sarcoidosis [44,45,46].

## 5. Materials and Method

### 5.1. Study Design

We chose a scoping review method due to the significant heterogeneity of the operationalization of the concept of nutritional status in the literature on sarcoidosis—including anthropometric indicators, body composition assessment, immunonutritional indices, biomarkers, and dietary data—this scoping review does not aim to quantitatively synthesize the impact of nutritional status on endpoints or compare the magnitude of effects between studies. Instead, the aim of the study was to systematically map the types of nutritional and metabolic exposures, the tools used to assess them, and the spectrum of clinical outcomes analyzed, and to identify research gaps resulting from the lack of standardized measurement procedures.

Scope reviews are a relatively new methodological approach. Currently, there are limited recommendations for choosing between a systematic review and a scoping review when synthesizing evidence, especially when the literature has not yet been comprehensively reviewed, is extensive, heterogeneous, or complex, and therefore not suitable for a more detailed systematic review [47]. Our scoping review was prepared in accordance with the methodological framework described in the Joanna Briggs Institute handbook for scoping reviews and based on the Preferred Reporting Items for Systematic Reviews and Meta-Analyses extension for Scoping Reviews (PRISMA-ScR) guidelines [11,48].

### 5.2. Inclusion and Exclusion Criteria

To identify key aspects of the impact of nutritional status on the development and survival of patients with sarcoidosis, we developed a research question that clearly defines the population, concept, and context of the review.

The inclusion criteria were as follows:

- Articles published between 2015 and 2025;
- Articles from all types of research;
- Articles with full-text access;
- Articles in any language;
- Adult patients diagnosed with sarcoidosis;
- Nutritional status and the impact of malnutrition on the development and prognosis of sarcoidosis.

Exclusion criteria included:

- Publications older than 10 years;
- Lack of full-text articles;
- Studies in which nutritional status or nutritional factors were not analyzed;
- Publications covering the pediatric population;
- Preclinical studies.

#### 5.2.1. Population

This scoping review included publications on adult patients (≥18 years of age) diagnosed with sarcoidosis, regardless of clinical form, duration, severity, or predominant organ involvement. Both pulmonary and extrapulmonary sarcoidosis cases were included, including those involving the skin, eyes, heart, and central nervous system, provided that the diagnosis was made in accordance with generally accepted clinical, radiological, and/or histopathological criteria.

Studies conducted in various healthcare settings, including outpatient and hospitalized populations, as well as population-based studies and registry studies, were eligible for analysis, with no restrictions on gender, ethnicity, or geographic region. No exclusion criteria related to current pharmacological treatment were applied, allowing the inclusion of untreated patients and those receiving glucocorticosteroids, immunosuppressive drugs, or biological therapies, thereby reflecting the clinical population’s actual heterogeneity.

Publications covering only the pediatric population (<18 years of age) and studies in which data on patients with sarcoidosis could not be clearly separated from analyses covering other inflammatory or granulomatous diseases were excluded from the review. These criteria were adopted to focus the study on the adult population of patients with sarcoidosis, in whom the impact of nutritional status on disease development, clinical course, and long-term prognosis has direct, practical clinical significance.

#### 5.2.2. Concept

The scope review included publications on the broadly defined nutritional status of patients with sarcoidosis. The concepts analyzed included both classic anthropometric indicators, such as body mass index, waist circumference, and body composition parameters, as well as malnutrition, obesity, and sarcopenia. Studies assessing dietary patterns and habits, macro- and micronutrient intake, and biomarkers related to nutrition and metabolism, including vitamin D and calcium concentrations and diet-related inflammatory markers, were included. Studies on nutritional or supplementation interventions were also included in the review if conducted in patients with sarcoidosis. They provided data relevant to mapping the relationship between nutritional status and the development, course, or prognosis of the disease.

#### 5.2.3. Context

Publications from any clinical and population setting were included, covering both outpatient and inpatient care, as well as cohort studies, registry-based analyses, and large databases. No geographical or institutional restrictions were applied, allowing inclusion of studies from diverse healthcare systems and populations with varied epidemiological backgrounds. This approach enabled comprehensive mapping of the available evidence, regardless of study location or healthcare model.

#### 5.2.4. Type of Study

For the sake of transparency, the source synthesis was divided into: primary studies (observational/interventional), which formed the basis for mapping the relationship between nutritional and metabolic exposures and outcomes in sarcoidosis, and secondary studies (e.g., narrative/clinical reviews), which were used solely to describe the biological background and interpret research directions, without giving them equal weight in the results table. This broad approach to study types was consistent with the nature of a scoping review, which aims to identify, classify, and describe the available literature, rather than to evaluate the effectiveness of specific interventions.

### 5.3. Search Strategy

The literature search strategy was designed systematically and reproducibly, in accordance with PRISMA-ScR recommendations, to identify publications on the relationship between nutritional status and the development, course, and prognosis of sarcoidosis. Two independent researchers searched the following electronic databases: PubMed, Scopus, Web of Science, Cochrane Library, EBSCO, and Google Scholar. The search covered publications from 2015 to 2025. The Safari search engine was used for the search. The following keywords were used: “sarcoidosis,” “nutritional status,” “nutrition,” “diet,” “dietary patterns,” “body mass index,” “obesity,” “malnutrition,” “sarcopenia,” “vitamin D,” “calcium metabolism,” “micronutrients,” and “metabolic factors.” Keywords and their combinations were entered using AND or OR. All publications were analyzed based on title and abstract to exclude irrelevant records. Any discrepancies were resolved through consultation with all three researchers, and at the end of the selection process, complete agreement was reached on the articles to be included. The initial search lasted until 1 October 2025, and the final search was conducted on 8 December 2025.

Only peer-reviewed articles published in indexed journals were included in the review; preprints and articles “in press” were not considered.

### 5.4. Extraction of Data

A data extraction form based on the JBI guidelines for scoping reviews [48] was used to incorporate key information from the studies. Data extraction, referred to in scoping reviews as “charting data” [49], was performed by two independent reviewers. The Population-Concept-Context (PCC) model was used to identify relevant studies. Information extracted from the studies included: first author, year, country, study design, study purpose, inclusion and exclusion criteria (PCC), results, and findings. The authors performed the extraction using Microsoft Excel.

### 5.5. Critical Appraisal Process

In accordance with the methodology of scoping reviews, the assessment of methodological quality/risk of systematic error of the included studies is not mandatory. It may be omitted when the aim is to map the scope and nature of the evidence and identify research gaps, rather than to assess the effect of interventions or the strength of evidence. Due to the lack of a formal quality assessment, this paper does not formulate causal conclusions or clinical recommendations based on the hierarchy of evidence; the relationships presented were interpreted as associations and descriptions of the scope of the literature [49].

### 5.6. Process for Including Publications in the Review

**Figure 2:**
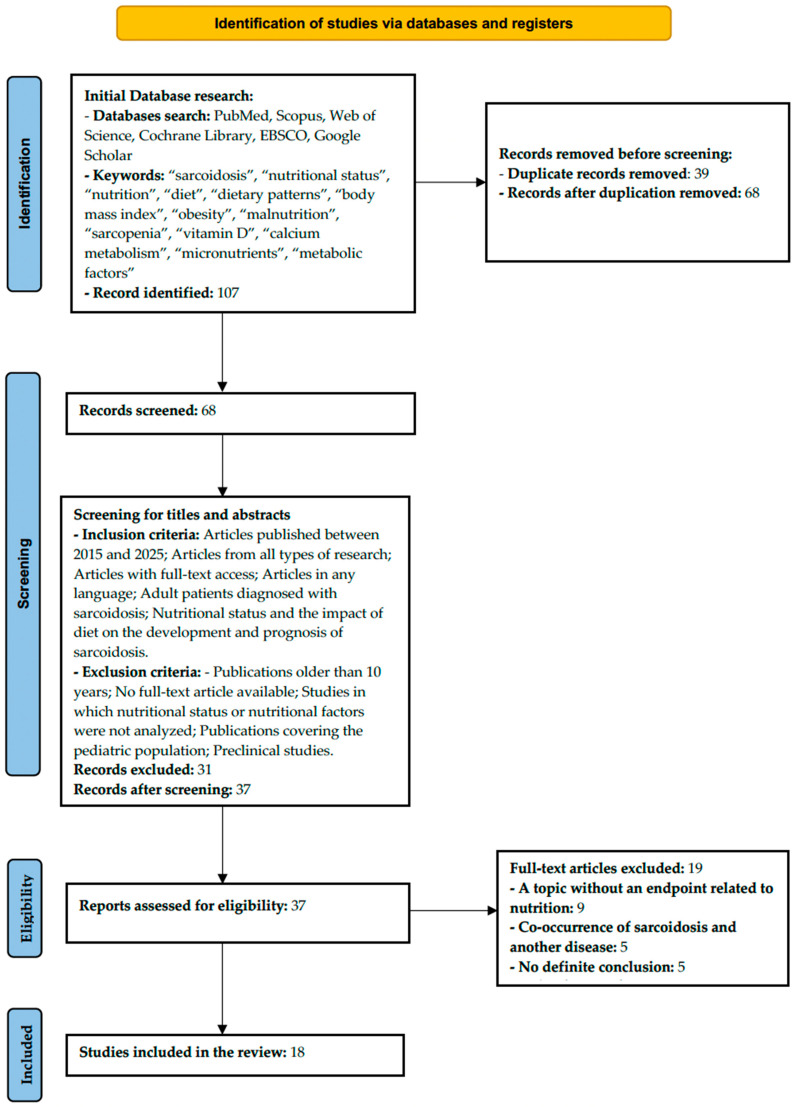
PRISMA 2020 flow diagram illustrating the study selection process.

As part of the scoping review, 107 records were initially identified in the databases (PubMed, Scopus, Web of Science, Cochrane Library, EBSCO, and Google Scholar). After removing 39 duplicates, 68 publications were selected for further analysis. After evaluating the titles and abstracts against the inclusion criteria, 31 articles were excluded, leaving 37 for further evaluation. Of these, 19 articles were rejected, mainly due to: lack of association between the endpoint and nutrition (*n* = 9), coexistence of another disease with sarcoidosis (*n* = 5), and lack of a clear conclusion (*n* = 5). Ultimately, 18 studies that met the eligibility criteria were included in the review (Figure 1). The included studies were conducted in the USA (*n* = 7), Turkey (*n* = 3), Poland (*n* = 4), India (*n* = 1), Japan (*n* = 1), France (*n* = 1), and China (*n* = 1). The analyzed studies included a variety of research designs, including prospective cohort studies, case–control studies, cross-sectional analyses, retrospective observational studies, and narrative and clinical reviews. The study populations included both large population cohorts and smaller patient groups with confirmed sarcoidosis. The data selection is presented in the PRISMA diagram in Figure 2.

Nutritional status assessment in the analyzed studies was based on various indicators and tools, including body mass index (BMI), bioimpedance analysis of body composition, biochemical parameters (calcium, phosphate, 25(OH)D, and 1,25(OH)_2_D concentrations), the CONUT scale (Controlling Nutritional Status score), and modeled sodium intake data. Endpoints included risk of sarcoidosis, disease activity, extrapulmonary involvement, recurrence, exercise capacity, quality of life, disorders of calcium and vitamin D metabolism, and risk of hypercalcemia.

For clarity, the results have been organized by domain: anthropometry and adiposity (e.g., BMI, weight gain), body composition (e.g., BIA), immunonutrition indices based on laboratory parameters (e.g., CONUT), nutrition/metabolism-related biomarkers (e.g., C-reactive protein, D-dimer, ferritin, homocysteine, triglycerides, insulin, glucose, cort BMI, weight gain), body composition (e.g., BIA), immunonutritional indices based on laboratory parameters (e.g., CONUT), biomarkers related to nutrition/metabolism (e.g., calcium-vitamin D metabolism), dietary factors based on intake estimates (e.g., modeled sodium intake). This approach reflects the range of available evidence but also highlights the lack of consistent, comparable methods for assessing nutritional status in sarcoidosis.

The results of the analyses indicate that overweight and obesity are significantly associated with an increased risk of developing sarcoidosis and may contribute to a more severe course of the disease and reduced exercise tolerance. Calcium and vitamin D metabolism disorders are a common clinical problem in patients with sarcoidosis, and vitamin D supplementation is associated with an increased risk of hypercalcemia and requires special caution. In addition, specific nutritional and metabolic parameters, such as sodium intake and nutritional status indicators, may be prognostically significant for disease activity and recurrence, although the available data are heterogeneous.

Detailed characteristics of the studies included in the scoping review, along with their key findings and limitations, are presented in Table 1 and Table S1.

Table 1 includes only primary studies. Secondary studies (narrative/clinical reviews) are listed separately in Table S1 and were used only to describe the background and identify hypotheses.

## 6. The Impact of Food on the Development of Sarcoidosis

Over the last decade, there has been a growing body of epidemiological data suggesting that nutritional status—in particular, overweight and obesity—may be associated with an increased risk of developing sarcoidosis, even though the etiology of the disease remains unclear. The most consistent findings concern adiposity (obesity) as a potentially modifiable risk factor. In contrast, evidence on other dimensions of nutrition (e.g., diet quality, micro- and macronutrients) is much rarer and more often indirect or ecological in nature.

The strongest evidence in the analyzed area comes from large cohort studies. The Black Women’s Health Study (BWHS), involving approximately 59,000 black women observed prospectively, showed a clear relationship between obesity and the incidence of sarcoidosis: compared to women with a BMI of 20–24 kg/m^2^, the risk was higher for both BMI 30–34 kg/m^2^ (IRR 1.54; 95% CI: 1.19–1.99) and especially for BMI ≥ 35 kg/m^2^ (IRR 1.90; 95% CI: 1.44–2.51). Importantly, a dose–response relationship was also observed for weight gain in adulthood: compared to individuals without significant weight gain, a weight increase of 10–19 kg was associated with an IRR of 1.32 (95% CI: 1.05–1.67), and weight gain of ≥20 kg with an IRR of 1.53 (95% CI: 1.22–1.91). These results reinforce the hypothesis that both current obesity and long-term weight dynamics may be important for disease initiation risk [60].

The relationship between obesity and the risk of sarcoidosis has also been confirmed in clinical control studies. A population-based study from Olmsted County (USA) (345 cases and 345 controls) found that, compared to individuals with normal/low BMI, obesity (BMI > 30 kg/m^2^) was associated with an OR of 2.54 (95% CI: 1.58–4.06), while overweight (BMI 25–29.9 kg/m^2^) did not reach statistical significance (OR 1.12; 95% CI: 0.72–1.75). Notably, after further adjustment for smoking and ethnicity, the association with obesity persisted (e.g., OR 2.48; 95% CI: 1.62–3.80), limiting the likelihood of a simple explanation by fundamental confounding factors [50].

In narrative reviews and expert analyses published in recent years, obesity is consistently identified as one of the best-documented non-genetic risk factors for sarcoidosis. It is emphasized that adipose tissue functions as an active endocrine and immune organ, and that chronic low-grade inflammation associated with obesity may dysregulate the immune response. Although these mechanisms remain largely hypothetical, their significance is consistent with epidemiological observations [35,61].

However, it should be noted that most available data concern the relationship between obesity and sarcoidosis incidence. At the same time, evidence on other aspects of nutritional status, such as qualitative malnutrition, body composition, or dietary patterns, is minimal. Clinical studies comparing patients diagnosed with sarcoidosis with healthy individuals show that patients are more likely to have a higher BMI and greater body fat mass. However, these studies are cross-sectional in nature and do not allow for clear cause-and-effect conclusions [41].

Considering epidemiological observations, the literature emphasizes the potential role of the immunometabolic axis: adipose tissue is an active endocrine-immune organ, and obesity is associated with chronic, low-grade inflammation and altered secretion of adipokines (including leptin) and pro-inflammatory cytokines, which can modulate innate and acquired immune responses. In a population study, Ungprasert et al. point to leptin as one of the plausible factors maintaining a pro-inflammatory immune response profile, potentially conducive to an environment that allows the formation and maintenance of granulomas, while noting that these are hypotheses that require further experimental verification [50].

Apart from adiposity, individual studies attempt to link dietary parameters with the burden of sarcoidosis at the population level. A time series analysis based on Global Burden of Disease data (1999–2019) showed a positive correlation between sodium intake and the incidence/prevalence of ILD, including pulmonary sarcoidosis; with very high sodium intake, the maximum effect was reported for incidence at RR 1.75 (95% CI: 1.61–1.91; lag 0) and for prevalence at RR 3.19 (95% CI: 2.24–4.53; lag 0) compared to the reference value. The authors point to the potential importance of diet-dependent modulation of inflammation. Still, it should be emphasized that this is an ecological study, subject to ecological bias and incapable of causal inference at the individual level. Therefore, these results should be interpreted as a signal requiring verification in individual (cohort/interventional) studies, rather than as evidence of a direct effect of sodium on the pathogenesis of sarcoidosis [53].

From an interpretative perspective, it is crucial that the available data—despite the consistency of the effect—remain largely observational; therefore, conclusions should be drawn with caution: the relationship shown is currently primarily an epidemiological association and still requires confirmation in mechanistic and interventional studies.

## 7. Nutritional Status and Its Impact on Survival and Prognosis in Sarcoidosis

Survival and prognosis in sarcoidosis are heterogeneous and depend on many factors, including the severity of organ involvement, the presence of pulmonary fibrosis, organ failure, and coexisting metabolic and nutritional disorders. General epidemiological data show that mortality rates are higher in patients with sarcoidosis compared to the general population. In a cohort study with a long follow-up period, significantly lower survival rates were reported in the group of patients with sarcoidosis compared to those without the disease: the 15-year survival rate was approximately 62% vs. 72% in the control group, and the hazard ratio (HR) for all-cause mortality in sarcoidosis was significantly increased (HR ≈ 1.8; 95% CI: 1.7–2.0; *p* < 0.001), even after adjusting for demographic and clinical risk factors [62].

Studies evaluating immunonutritional indicators provide important information regarding prognosis. A retrospective analysis of 156 patients with sarcoidosis showed that although the classic CONUT malnutrition index did not significantly differentiate between groups in terms of disease course, ACE/lymphocytes was significantly associated with both extrapulmonary involvement and the risk of disease recurrence, both of which are considered unfavorable prognostic factors in clinical practice. These results suggest that components of nutritional and immune status may be necessary in identifying patients with a poorer prognosis, even if they do not directly translate into hard endpoints such as survival [51].

Studies show that 25-hydroxyvitamin D (25(OH)D) deficiency is widespread in the sarcoidosis patient population, with rates of 60–80% across various clinical and epidemiological cohorts. At the same time, controlled clinical trials have shown that sarcoidosis is associated with frequent elevation of the active form of 1,25(OH)_2_D, resulting from extrarenal 1α-hydroxylase activity in granuloma macrophages, which can lead to hypercalcemia and hypercalciuria despite low levels of 25(OH)D [44,63].

In a study by Kiani et al. analyzing 80 patients with sarcoidosis, it was shown that 25(OH)D deficiency was statistically significantly associated with more advanced radiological lung involvement (stage 2–4 in the Scadding system) compared to patients with normal vitamin D levels (72% vs. 50%; *p* = 0.039) and that a higher percentage of patients with deficiency had a chronic rather than acute course of the disease (55% vs. 28%; *p* = 0.012). These results suggest that lower 25(OH)D levels are correlated with poorer clinical indicators and a potentially more severe course of the disease [64].

The biological role of vitamin D in modulating the immune system is well documented in the context of inflammatory and autoimmune diseases. Vitamin D can influence macrophage and T lymphocyte function and cytokine regulation, potentially affecting the inflammatory activity of granulomatous disease. Despite these mechanisms, there are no clear clinical studies evaluating the therapeutic effect of correcting 25(OH)D deficiencies on survival or disease progression in sarcoidosis, and the observed effects of supplementation are controversial due to the risk of hypercalcemia [65,66].

In a retrospective study, Sodhi and Aldrich showed that vitamin D supplementation in patients with sarcoidosis was associated with a significantly higher risk of hypercalcemia, which is a potential factor worsening prognosis, especially in patients with renal failure. Although these data do not directly relate to survival, they emphasize the importance of individual assessment of nutritional status and the safety of supplementation in the context of long-term prognosis [45].

There is a lack of broad prospective analyses in the literature assessing the impact of qualitative malnutrition (e.g., sarcopenia, micronutrient deficiencies) on survival in sarcoidosis. Nevertheless, analogies with other chronic inflammatory diseases indicate that reduced lean body mass and metabolic dysfunction are associated with poorer clinical outcomes. Therefore, further studies are needed to evaluate specific nutritional profiles, beyond BMI, as predictors of prognosis.

## 8. Discussion

This scoping review synthesizes current evidence on the relationship between nutritional status and the development and course of sarcoidosis, with a particular focus on immunometabolic mechanisms. Available epidemiological data clearly indicate that overweight and obesity are consistently associated with an increased risk of developing sarcoidosis. Still, this relationship cannot be interpreted solely in terms of excess body weight.

It should be emphasized that most studies were observational, and no formal assessment of systematic error risk was conducted in the scoping review. Therefore, the presented relationships should be treated as associations and premises for further research, rather than as a basis for inferring causality or formulating clinical recommendations.

Obesity is now recognized as a chronic low-grade inflammatory condition in which adipose tissue acts as an active endocrine and immune organ. Adipocytes and immune cells infiltrating adipose tissue produce numerous pro-inflammatory cytokines (including TNF-α and IL-6) and adipokines, such as leptin, which promote polarization of the immune response towards Th1 and Th17 cells and inhibit regulatory T cell activity. Since the pathogenesis of sarcoidosis is characterized by the dominance of the Th1 response and macrophage activation, leading to the formation of non-caseating granulomas, the immune environment characteristic of obesity is a biologically plausible mechanism for initiating or maintaining the disease [25,35,60,61].

This hypothesis is supported by the results of extensive cohort studies, which have demonstrated a dose–response relationship between BMI, weight gain in adulthood, and the risk of developing sarcoidosis, independent of smoking and other confounding factors. Although the available data are observational and do not allow for clear cause-and-effect conclusions, their consistency and compatibility with current immunological knowledge support the critical role of immunometabolic disorders [37,50].

In addition to quantitative assessment of body weight, increasing importance is being attached to the quality of the diet and its impact on the gut-immune axis. A diet rich in fiber promotes the production of short-chain fatty acids (SCFA), which have anti-inflammatory effects by promoting the differentiation of regulatory T cells and limiting macrophage activation [10,16,18]. In contrast, a highly processed diet, rich in sodium and low in fiber, is associated with intestinal dysbiosis, increased intestinal barrier permeability, and exacerbation of systemic inflammatory signals [23,24]. Although direct studies of the gut microbiota in sarcoidosis are limited, ecological data suggesting a link between high sodium intake and a greater burden of pulmonary sarcoidosis suggest a potential role for dietary factors in modulating the disease phenotype [53,60,67].

An essential addition to the discussion on SCFA and the gut-immune axis is the emphasis on data already supporting the microbiome’s involvement in sarcoidosis. However, these data come predominantly from analyses of the respiratory and circulatory microbiota rather than from intestinal studies. BAL-based studies have shown differences in the composition of the lung microbiota in patients with sarcoidosis (including enrichment with *Atopobium* and *Fusobacterium*), suggesting that respiratory tract dysbiosis may contribute to an environment conducive to maintaining a granulomatous response [30,31]. In addition, blood microbiome analyses have described characteristic patterns of abundance of selected bacterial species in patients with sarcoidosis, supporting the hypothesis of chronic exposure to microbial stimuli that may sustain innate immune activation [32]. At the same time, reports link the gut microbiota profile to the immune signature (including axes related to heme metabolism and monocyte activation), providing a potential mechanistic bridge between diet, microbiota, metabolites (including SCFA), and the perpetuation of inflammation [33]. At the same time, it should be emphasized that there are currently no interventional studies in sarcoidosis that clearly demonstrate that dietary modification to increase SCFA production results in a clinically significant reduction in disease activity, which remains a key research gap.

Body composition is also an essential factor in nutritional status in sarcoidosis. Clinical studies have shown that increased fat mass and unfavorable body composition are associated with reduced exercise capacity and muscle strength, which are important clinical indicators of disease burden and quality of life in patients [41,68].

A distinctive feature of sarcoidosis is abnormal vitamin D and calcium metabolism. Extrarenal activation of 1α-hydroxylase in granuloma macrophages leads to increased concentrations of active 1,25(OH)_2_D, which may result in hypercalcemia and hypercalciuria even at low levels of 25(OH)D. Consequently, vitamin D supplementation, commonly recommended in the general population, may increase the risk of metabolic complications in patients with sarcoidosis and requires individual assessment and close monitoring [44,45,52,65].

In summary, available evidence indicates that nutritional status is directly correlated with sarcoidosis through complex, interrelated immunometabolic, inflammatory, and metabolic mechanisms. Obesity and unfavorable dietary patterns are consistently associated with the risk of developing the disease, while data on prognosis and survival remain limited and indirect. Further prospective and interventional studies are needed to assess whether targeted nutritional strategies and lifestyle modifications can beneficially influence the course and long-term outcomes of sarcoidosis treatment.

### Methodological Limitations and Interpretation of Results

Nutritional status is a complex, multidimensional concept and should therefore not be interpreted as a single, independent exposure but rather as a marker of coexisting metabolic, behavioral, and environmental factors. Most of the studies analyzed used simplified measures, mainly BMI, which do not reflect body composition or metabolic heterogeneity, which is particularly important in sarcoidosis. In addition, there is a high risk of confounding factors, such as glucocorticosteroid treatment, limited physical activity, or comorbidities, as well as the possibility of reverse causality. Due to the observational nature of the available data and the lack of a formal assessment of study quality, the results presented should be interpreted as mapping associations and generating research hypotheses, rather than causal evidence.

## 9. Research Gaps and Clinical Implications

Evidence mapping reveals several key gaps:

- Lack of standardization in measuring nutritional status (different tools and different constructs), which hinders comparison and synthesis;
- A shortage of prospective studies with repeatable exposure measurements and control of confounding factors (including GCS treatment, physical activity, and comorbidities);
- A limited number of studies used measures of diet quality and objective assessment of body composition rather than BMI alone;
- Lack of interventional studies assessing whether dietary modifications or targeted metabolic interventions translate into clinically relevant endpoints; lack of data on sarcoidosis-specific mortality in relation to nutritional status.

## 10. Directions for Future Research

The results of this scoping review indicate significant research gaps in the relationship between nutritional status and the development, course, and prognosis of sarcoidosis. Despite a growing body of epidemiological studies suggesting a link between excess body weight and the risk of developing the disease, the available data are predominantly observational and limited to selected populations, making it difficult to draw causal conclusions.

Future studies should focus on prospective cohorts of patients with newly diagnosed sarcoidosis, with long-term follow-up including hard endpoints such as disease progression, hospitalizations, critical organ involvement, and overall and disease-specific survival. Standardization of nutritional assessment beyond BMI alone, including body composition analysis, malnutrition risk screening tools, immunonutritional markers, and diet quality, is also crucial.

Mechanistic analyses are also an essential direction for further research, as they would allow for a better understanding of the potential role of the immunometabolic axis in the pathogenesis of sarcoidosis, including the importance of adipose tissue as an active endocrine organ and its impact on the granulomatous response. In the clinical context, particular attention should be paid to studies on the safety and efficacy of nutritional interventions, especially vitamin D supplementation, that consider the risk of hypercalcemia and individual metabolic differences in patients with sarcoidosis.

Studies involving diverse ethnic and geographic populations are also needed to assess whether the observed associations between nutritional status and sarcoidosis are universal or modified by environmental, socioeconomic, and cultural factors.

Future studies should be characterized by higher methodological quality and the use of uniform standards for reporting nutritional and metabolic exposures.

## 11. Conclusions

To ensure clarity, the available study findings were classified according to whether the analyzed nutritional factors acted as risk factors for the development of sarcoidosis or as prognostic indicators related to disease course, relapse, functional capacity, quality of life, and metabolic complications.

An analysis of the literature from the past decade indicates that nutritional status—particularly excess body weight and obesity—represents the most consistent and reproducible nutritional factor associated with an increased risk of developing sarcoidosis. This association has been confirmed in cohort and population-based studies, although the underlying biological mechanisms remain incompletely understood.

Regarding disease course and prognosis, the available evidence is largely indirect. It suggests that selected components of nutritional status, including body composition, immunonutritional markers, and disturbances in calcium–vitamin D metabolism, may influence disease severity, relapse risk, and the occurrence of organ-related complications in sarcoidosis. However, there are currently few studies that allow a definitive determination of the impact of nutritional status on survival in patients with sarcoidosis.

In light of the current evidence, the assessment of nutritional status should be regarded as an important component of comprehensive care for patients with sarcoidosis, particularly in the context of monitoring body weight, physical performance, and the safety of vitamin D supplementation, which requires an individualized approach. Nevertheless, the available data are insufficient to support the formulation of universal clinical recommendations.

It should be emphasized that the existing evidence is limited by the predominantly observational nature of the studies, small sample sizes in many clinical cohorts, and limited population diversity, with a predominance of studies conducted in the United States. Interventional studies evaluating the impact of dietary modifications on the course of sarcoidosis are currently lacking.

In conclusion, this scoping review identifies key gaps in the existing literature and highlights directions for future research, underscoring the need for well-designed prospective and interventional studies to clarify the role of nutritional status as a potentially modifiable factor influencing the development and course of sarcoidosis.

## Figures and Tables

**Table 1:** Characteristics of primary studies included in the scoping review.

Author (Year)	Country	Research Design	Population	Nutritional Status Assessment	Outcomes Related to Sarcoidosis	Key Findings	Main Limitations	Source
Dumas et al. (2017)	USA	Prospective cohort study	59,000	BMI, weight gain	Incidence of sarcoidosis	Overweight and obesity are associated with an increased risk of disease	Female-only population; observational nature	[37]
Ungprasert et al. (2016)	USA	Nested case–control	345 cases/345 controls	BMI categories; smoking	Risk of sarcoidosis	Obesity (BMI ≥ 30) associated with higher sarcoidosis risk (OR ~ 2.5)	Retrospective; limited ethnic diversity	[50]
Tunç et al. (2025)	Türkiye	Prospective case–control	31 sarcoidosis/24 controls	BMI, bioimpedance (fat and lean mass)	Exercise capacity, muscle strength, lung function	Higher BMI and fat mass; nutritional status associated with reduced exercise capacity	Small sample; early-stage pulmonary sarcoidosis only	[41]
Canoglu et al. (2025)	Türkiye	Retrospective observational	156 patients	CONUT score (albumin, lymphocytes, cholesterol)	Extrapulmonary involvement, disease recurrence	CONUT not predictive; ACE/lymphocyte ratio associated with recurrence	Retrospective analysis	[51]
Gwadera et al. (2023)	Poland	Cross-sectional	58 patients/25 controls	Serum calcium, phosphate, 25(OH)D, 1,25(OH)_2_D	Disease activity, fatigue, quality of life	Phosphate is associated with QoL; calcium is not a marker of activity	Single time point; seasonal variation in vitamin D levels	[52]
Sodhi and Aldrich (2016)	USA	Retrospective case-control	196 sarcoidosis/196 controls	Vitamin D supplementation practices	Hypercalcemia risk	Vitamin D supplementation doubled risk of hypercalcemia	Retrospective	[45]
Hu et al. (2024)	China	Ecological time-series analysis	Population-level data	Dietary sodium intake (modeled)	Incidence and severity of pulmonary sarcoidosis	Higher sodium intake is associated with an increased burden of disease	Ecological design; outcome not sarcoidosis-specific	[53]
Betlejewska et al. (2020)	Poland	Case study	1 patient (sarcoidosis)	Vitamin D supplementation; 25(OH)D, Ca; clinical context of granulomatous disease	Hypercalcemia, acute kidney injury after supplementation	Oversupply of vitamin D in sarcoidosis can precipitate hypercalcemia and AKI; highlights need for monitoring in granulomatous disease.	Single case; not generalizable; observational	[54]
Robertson et al. (2017)	USA	Case report + narrative review	1 patient (steroid-treated sarcoidosis)	High-dose vitamin D supplementation (context of bone protection); calcium/vitamin D metabolism	Symptomatic hypercalcemia after vitamin D	High-dose vitamin D can trigger symptomatic hypercalcemia in sarcoidosis; provides management considerations for supplementation.	Case-based evidence; narrative review; no survival/prognosis endpoints	[55]
Chandran et al. (2022)	India	Case report + literature review	1 patient (systemic sarcoidosis; severe hypercalcemia)	Vitamin D deficiency treatment regimen (60,000 IU weekly); Ca, PTH, 1,25(OH)_2_D	Severe hypercalcemia; diagnostic workup confirming sarcoidosis; response to steroids	Severe hypercalcemia may follow vitamin D repletion in sarcoidosis; suggests cautious, low-dose supplementation with monitoring.	Single case; context-specific dosing practices	[56]
Mio et al. (2022)	Japan	Case report	1 patient (sarcoidosis on follow-up)	Cholecalciferol supplementation; 25(OH)D_3_ and 1,25(OH)_2_D_3_ levels; Ca	Hypercalcemia and acute kidney injury after 1 year of supplementation	Vitamin D supplementation can worsen hypercalcemia in sarcoidosis via increased conversion to calcitriol; recommends careful monitoring.	Single case; no incidence/survival endpoints	[57]
Besnard et al. (2023)	France	Prospective survey (conference abstract)	75 French internists (responses)/practice patterns	Vitamin D/calcium supplementation practices; dietary calcium advice (dairy intake) depending on calcemia/25(OH)D	Clinical management practices related to bone health and calcium disorders in sarcoidosis on steroids	Marked heterogeneity in vitamin D/calcium supplementation; dietary calcium advice used by a subset; calls for guidance and monitoring.	Abstract-level data; not patient outcomes; response bias	[58]
Işık et al. (2021)	Turkey	Cross-sectional study	253 sarcoidosis patients	CONUT score, Prognostic Nutritional Index (PNI), CRP/albumin ratio, NLR	Association with metabolic syndrome (MetS) in sarcoidosis	Any degree of malnutrition by CONUT was associated with higher prevalence of MetS; NLR/CONUT suggested as early-stage markers for MetS risk in sarcoidosis.	Single-center; cross-sectional; does not assess incidence or survival	[59]

BMI—body mass index; OR—odds ratio; CONUT—Controlling Nutritional Status score; ACE—angiotensin-converting enzyme; QoL—quality of life; 25(OH)D—25-hydroxyvitamin D; 1,25(OH)_2_D—1,25-dihydroxyvitamin D; Ca—calcium; PTH—parathyroid hormone; AKI—acute kidney injury; PNI—Prognostic Nutritional Index; CRP—C-reactive protein; NLR—neutrophil-to-lymphocyte ratio; MetS—metabolic syndrome; IU—international units.

## Data Availability

No new data were created or analyzed in this study.

## Supplementary Materials

The following supporting information can be downloaded at: https://www.mdpi.com/article/10.3390/nu18020209/s1, Table S1: Secondary studies on the relationship between nutritional status and sarcoidosis.

## Abbreviations

ACE	Angiotensin-Converting Enzyme
ATS	American Thoracic Society
BMI	Body Mass Index
CONUT	Controlling Nutritional Status Score
CRP	C-Reactive Protein
ERS	European Respiratory Society
GCS	Glucocorticosteroids
GBD	Global Burden of Disease
IL	Interleukin
JBI	Joanna Briggs Institute
NF-κB	Nuclear Factor Kappa B
OR	Odds Ratio
PCC	Population–Concept–Context
PRISMA	Preferred Reporting Items for Systematic Reviews and Meta-Analyses
PRISMA-ScR	Preferred Reporting Items for Systematic Reviews and Meta-Analyses Extension for Scoping Reviews
QoL	Quality of Life
SAPF	Sarcoidosis-Associated Pulmonary Fibrosis
SAPH	Sarcoidosis-Associated Pulmonary Hypertension
SCFAs	Short-Chain Fatty Acids
TNF-α	Tumor Necrosis Factor Alpha
UPF	Ultra-Processed Foods
USA	United States of America
25(OH)D	25-Hydroxyvitamin D (Calcifediol)
1,25(OH)_2_D	1,25-Dihydroxyvitamin D (Calcitriol)
