# Supplementary material for: The Impact of Nutritional Status on Survival and Development of Sarcoidosis: A Scoping Review of Current Evidence and Research Gaps
Source: Nutrients. 2026 Jan 9;18(2):209. doi: 10.3390/nu18020209 (PMC12844605; doi:10.3390/nu18020209)
Supplement: Supplementary file 1 [file nutrients-18-00209-s001.zip › nutrients-4080028-supplementary.pdf]

Table S1. Secondary studies on the relationship between nutritional status and sarcoidosis

**Author (Year)**	**Country**	**Research design**	**Population**	**Nutritional status assessment**	**Outcomes related to sarcoidosis**	**Key Findings**	**Main limitations**	**Sourse**
Cozier (2018)	USA	Narrative review	Not reported	Obesity, adiposity (conceptual)	Risk and worse course of the disease	Obesity is proposed as an immunometabolic contributor to sarcoidosis development and persistence	Narrative design; no primary data	[35]
Cozier (2022)	USA	Focused clinical review	Not reported	BMI, central obesity	Sarcoidosis risk	Summarizes accumulating evidence linking obesity with increased sarcoidosis risk	Review article; heterogeneity of cited studies	[61]
Gwadera et al. (2019)	Poland	Narravive-clinical review	Not reported	Vitamin D and calcium metabolism	Hypercalcemia, disease course	Highlights the disturbed calcium–vitamin D axis in sarcoidosis	Review; no original data	[65]
Gianella et al. (2020)	USA	Narrative review	Not reported	Vitamin D metabolism and supplementation	Hypercalcemia risk	Vitamin D acts as a "double-edged sword" in sarcoidosis	Review; lack of interventional trials	[44]
Bieda et al. (2025)	Poland	Narrative review (literature analysis)	N/A (review of 107 full-text articles up to Nov 2024)	Vitamin D metabolites; supplementation risk/benefit in sarcoidosis	Discussion of disease activity, progression, fracture risk; hypercalcemia risk with supplementation	Argues vitamin D supplementation may be considered with individualized risk–benefit assessment and close monitoring due to hypercalcemia risk.	Narrative synthesis; not primary data; language may be English despite Polish journal	[66]

BMI – body mass index; USA – United States of America; N/A – not applicable; Vit. D - vitamin D; Ca-calcium
